# Manifestation of leech infestation as severe gastrointestinal bleeding in a 3-year-old boy: a case report and review of the literature

**DOI:** 10.1186/s12887-022-03778-1

**Published:** 2022-12-08

**Authors:** Hamid Reihani, Ali Ghanei-Shahmirzadi, Sara Salehi, Narges Ansari-Charsoughi, Fereshteh Karbasian, Mohammad-Hadi Imanieh

**Affiliations:** 1 Student Research Committee, School of Medicine, Shiraz University of Medical Sciences, Shiraz, Iran; 2 Department of Pediatric Gastroenterology, Shiraz University of Medical Sciences, Shiraz, Iran; 3 Gastroenterohepatology Research Center, Shiraz University of Medical Sciences, Shiraz, Iran

**Keywords:** Leeches, Gastrointestinal hemorrhage, Melena, Hematemesis

## Abstract

**Background:**

Leeches are a class of hermaphroditic parasites that can attach to various body parts and start sucking blood. Gastrointestinal (GI) bleeding due to leeches is a rare phenomenon that is more common in less developed countries. Common symptoms include melena, hematemesis, pallor, weakness, and fatigue. Due to the similar symptoms of this issue to the main differential diagnoses of GI bleeding in pediatrics, such as diarrhea, constipation, diverticulitis, esophagitis, and anal fissures, it is challenging to differentiate it from the rest.

**Case presentation:**

We present a three-year-old boy who was transferred to our center with hematemesis, tarry stool, and a drop in hemoglobin level. He finally was diagnosed with a leech in his stomach.

**Conclusions:**

In less developed counties, the inability to reach safe drinking water, swim in lakes or springs, and inadequate awareness of public health information among individuals can be risk factors for leech infestation.

## Background

When it comes to serving as a physician in developing countries, some extremely rare differential diagnoses should be considered. Leech infestation is one of those rare conditions which can be the source of gastrointestinal (GI) bleeding. Leeches are a group of hermaphroditic parasites that can attach to several areas in the human body and start sucking blood [1]. They surprisingly can engorge up to 890% of their body weight [2]. Furthermore, they have some anticoagulant-like enzymes in their saliva that can lead to severe bleeding after infestation, which in complicated cases can also lead to the patient’s death [3, 4]. They can involve various parts of the GI tract and the respiratory system, including the nasopharynx, oral cavity, larynx, esophagus, and rectum [5, 6]. GI bleeding, which is caused by leech infestation, can manifest itself in different patterns, such as epistaxis, hematemesis [7], melena [8], and fresh rectal bleeding [9]. Here we present a 3-year-old boy who transferred to our center with tarry stool and bloody vomiting. He finally was diagnosed with a leech in his stomach.

## Case presentation

**Fig. 1:**
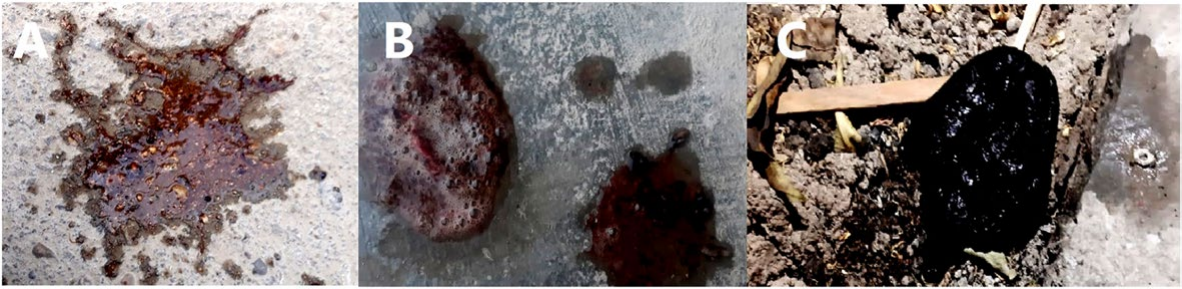
Photos taken of the patient’s hematemesis (**A, B**) and melena (**C**) by the parents

**Fig. 2:**
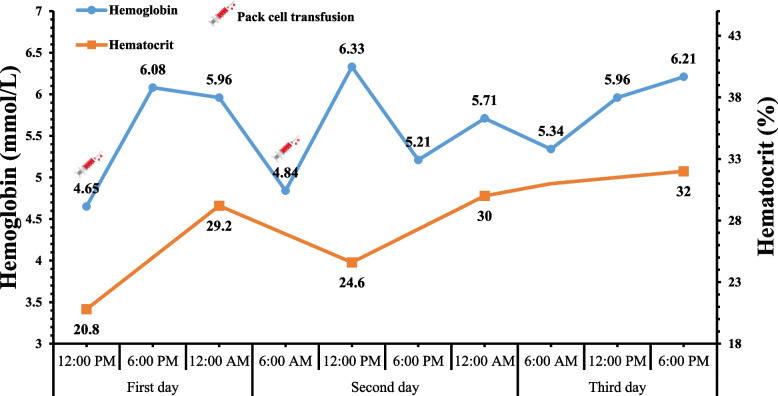
Changes in hemoglobin and hematocrit levels during hospitalization

A 3-year-old boy was transferred to our emergency department, which is a referral center, from a primary care hospital near his house. Tarry stool from a week ago and hematemesis that started yesterday were his main complaints. According to the patient’s mother, his stool began to darken last week, but they didn’t consider it an important issue. Then, 2 days prior to admission, he suddenly vomited blood and was rushed to the nearest local hospital (Fig. 1). At the time, he had a drop in hemoglobin level, which was followed by a blood transfusion, and he also started on ondansetron and pantoprazole. The patient’s hemoglobin level was at acceptable levels following transfusion; however, a control complete blood count analysis at 18 hours’ post-admission showed a decrease in hemoglobin level. Therefore, after having packed red blood cell transfusion, he was referred to our hospital for further investigation of the underlying disease. The laboratory course of hemoglobin and hematocrit is shown in Fig. 2.

First evaluation of the patient at the emergency department revealed that he had a blood pressure of 110/70 mmHg, a pulse rate of 120 beats per minute, a respiration rate of 18, and an oxygen saturation of 98%. His general appearance was slightly pale, but he did not have any complaints of vomiting or diarrhea. There was no evidence of petechiae, purpura, or bruising on his skin. Oropharynx was without inflammation or lesion, and there was no nasal polyp, obstruction, or abnormal discharge from the nasal mucosa. His parents did not declare any history of trauma or bleeding diathesis. Furthermore, the liver and spleen were not palpable on abdominal examination. The digital rectal examination revealed no anal lesion; however, melena was clearly visible. The patient’s medical history revealed that he was diagnosed with Gastroesophageal reflux disease (GERD) at the age of two. He was on omeprazole 10 mg orally once a day, but had stopped taking it 2 months prior to admission.

**Fig. 3:**
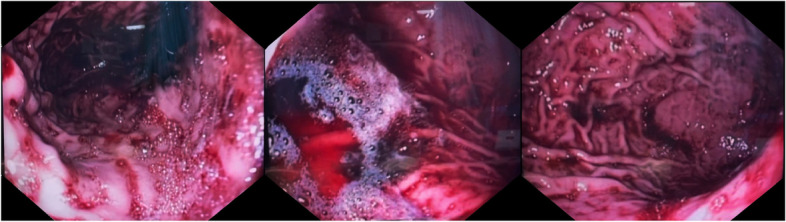
Stomach was full of fresh blood and was not entirely visible for finding the underlying cause

**Fig. 4:**
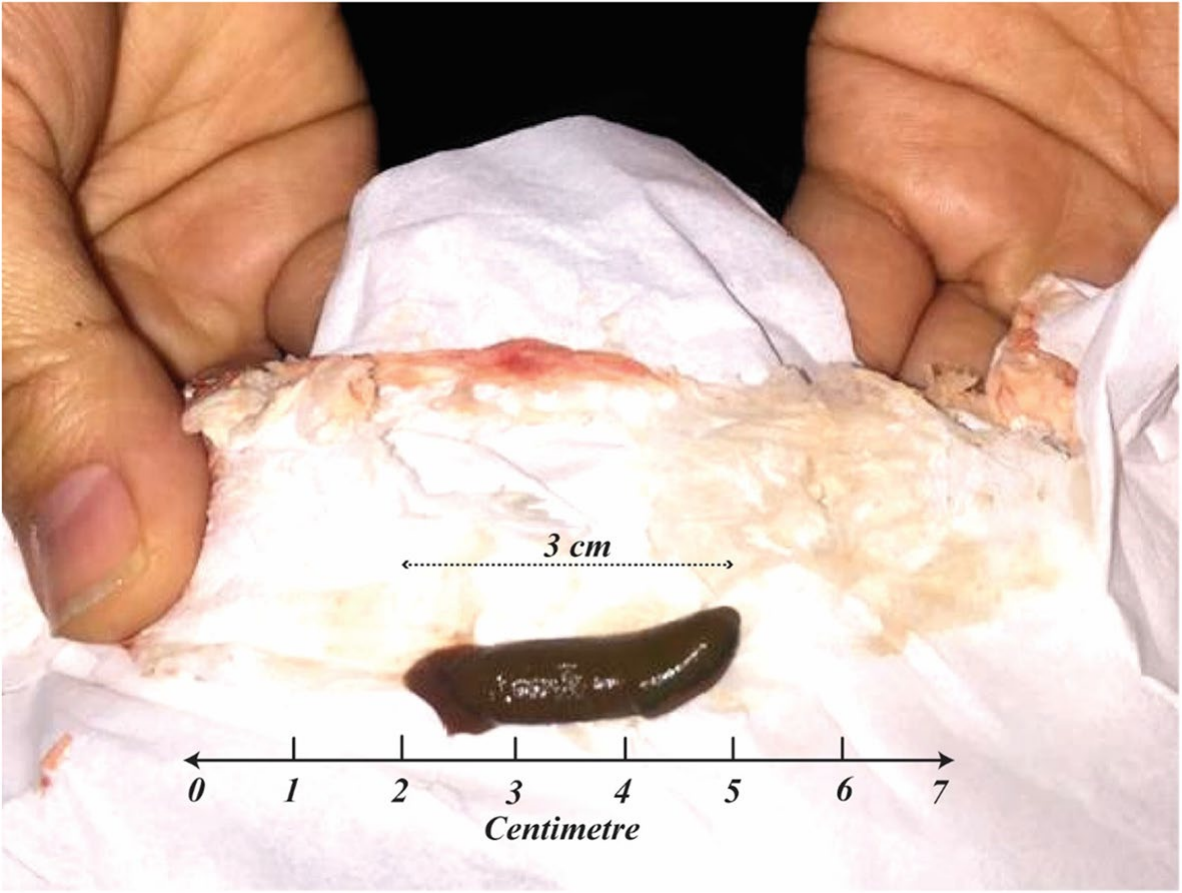
A leech removed from the patient’s stomach after vomiting

In our department, the patient’s laboratory tests were normal, and his hemoglobin level was 6.33 mmol/L, so there was no need to inject the packed cell again. The patient’s coagulation parameters were also in the standard range. However, he was scheduled for an upper endoscopy due to the history of hemoglobin drop during his previous hospitalization. Endoscopy reported the esophagus to be completely intact. Still, the stomach was not entirely visible due to the large volume of fresh blood, and the exact site of the bleeding was not localized (Fig. 3), so re-endoscopy was recommended to find the bleeding cause. The next day, while the patient was preparing for re-endoscopy, his mother noticed slight bleeding from her son’s mouth, along with his complaint of severe nausea. He then vomited a three-centimeter-long leech, shown in Fig. 4. Afterward, he reported a history of swimming in a spring near his home and drinking water there. Eventually, he was monitored for 24 hours, and his hemoglobin status was stable. Therefore, he was discharged in good general condition with the essential recommendations to his parents.

## Discussion and conclusions

Leeches are a large group of hematophagous hermaphroditic ectoparasites with many different species like sand leeches or aquatic leeches [10]. In some developing countries where people may use ponds or sewages water, it’s possible to get affected by aquatic leeches. For the most part, leeches’ main target is nasopharyngeal area [11]. But in some cases, it was reported to affect the rectum or esophagus [6, 10]. Due to the variety of leech infestation sites, this disease manifests itself with different symptoms such as epistaxis [8], hematemesis [7, 12], melena, rectal bleeding [6], and even cough [7]. For a comprehensive review of this topic, we conducted an advanced search of the PubMed/Medline, Scopus, and Web of Science databases using keywords and Mesh terms (“leeches” [Mesh], leech, hirudinea, “gastrointestinal Hemorrhage”[Mesh], hematochezia, hematemesis, melena), which are presented in Table 1.

**Table 1:** Characteristics of 11 included Case reports

Author, year	Age, gender	Country	Symptoms at presentation	Leech binding-location	Probable source	Hemoglobin (g/dl)	Endoscopy/Colonoscopy/Laryngoscopy report
Tilahun et al. 2020 [6]	4, Male	Ethiopia	Rectal bleeding, vomiting	Rectum	Small river near their house	NR	NR
Narayan et al. 2017 [13]	18, Male	India	Rectal bleeding, tenesmus	Rectum	Water logged field	4.96	presence of an ulcer of size 0.5 cm × 1 cm with hooklets and the head of leech attached at 10 cm from anal verge
Mamoudou et al. 2015 [14]	3, Male	Niger	Hematemesis, melena, epigastralgia	Behind the glottis	Drinking freshwater	5.15	endoscopic exploration revealed a normal-appearing mucosa
Kani et al. 2014 [12]	72, Male	Turkey	Hematemesis	Hypo-pharyngeal	Drinking water from an open trough	7.26	Several crater-type lesions located in oropharynx, tongue, esophagus, stomach
Koraichi et al. 2014 [8]	3, Female	Morocco	Melena, epistaxis	Nasopharynx	Drinking freshwater	4.34	Nasopharynx bleeding, small red mass in the pharynx.
Abuhandan et al. 2012 [15]	5, Male	Turkey	Epistaxis, hematemesis	Oropharynx	NR	4.59	NR
Rafeey et al. 2012 [16]	9, Male	Iran	Hematemesis, fever	Nasopharynx	Bathed in a pond	7.26	Endoscopy: normal, detected with ENT examination
Al et al. 2011 [9]	33, Male	Turkey	Rectal bleeding, anorectal discomfort	Rectum	Swimming in a contaminated pool	6.21	Dark brown, smooth foreign body just above the internal hemorrhoid swelling
Taskesen et al. 2009 [7]	1, Male	Turkey	Cough, coryza, epistaxis, hematemesis	Nose	Deep well near the family home	2.79	NR
Basu et al. 2004 [17]	4, Female	India	Rectal bleeding	Large bowel	NR	4.34	No active bleeding site was seen
Demirören et al. 2003 [5]	3, Male	Turkey	Exhaustion, pallor, hematemesis, melena	Oropharynx	Pool near the family home	1.86	NR
Raj et al. 2000 [18]	68, Female	Malaysia	Rectal bleeding, sensation of wanting to defecate	Rectum	NR	3.97	Fresh blood in the rectum and sigmoid colon
Iraqi et al. 1999 [19]	1.5, Male	Morocco	Hematemesis, dyspnea	Laryngopharyngeal	Bathed in fresh water	4.96	Nothing pathological in the digestive tract
Estambale et al. 1992 [20]	3, Male	Kenya	Cough, hematemesis, epistaxis	Not sure	NR	2.61	NR
el-Awad et al. 1990 [21]	10, Male	Saudi Arabia	Hematemesis, melena, epistaxis	Posterior pharyngeal wall	Bathed in ponds and sometimes drank water from them	Low	Did not show any abnormality
Singh et al. 1979 [22]	3, Male	Kabul	Cough, dyspnea, hematemesis	Not sure	Pond which is heavily infested with leeches	NR	NR

*NR* not reported

According to the collected studies, the majority of them occurred in children [5–8, 14–17, 19, 20, 22], which can highlight the role of parents in monitoring their children’s high-risk behaviors because the child does not understand the difference between healthy and non-drinking water. On the other hand, we surveyed the study countries and found that almost all were in low- or lower-middle-income countries with less access to clean water and less public health information [6, 13, 14, 16, 20, 22]. Finally, the source of this unhealthy water is usually reported springs, natural lakes, and rivers [5, 6, 12, 16, 19, 22], which are primarily located in rural areas and far from cities, and in some reports, just swimming in such waters and not even drinking from them can increase the probabilities of catching leech infestation.

The most exciting aspect of our case is the possibility of leech infestation in our patient’s stomach, which was not reported in other studies summarized in Table 1. In our physical examination, the nasopharynx was clear and without any lesions or bleeding; plus, endoscopy did not reveal any lesions in the esophagus and duodenum. Moreover, his mother detected the parasite in his vomiting contents. These clues have led us to believe leech infestation in the stomach is the most probable scenario in our case. Our rationale for this assumption is the long-term usage of proton pump inhibitors (PPIs) by the patient, which led to the modification of the patient’s gastric acid production, made him vulnerable to leech implantation in his stomach. Because gastric acid plays an essential role against parasitic diseases such as *Diphyllobothrium latum*, *Giardia lamblia*, and Strongyloides stercoralis [23, 24].

In conclusion, leech infestation is one of the rare reasons for GI bleeding, mainly reported in less developed countries where people cannot reach safe drinking water. Therefore, a history of consuming non-potable water or swimming in ponds or springs in cases where parasites are suspected is a must, especially in children, due to their less awareness of the health issues.

## Data Availability

Data of the patient can be requested from the authors. Don’t hesitate to get in touch with the corresponding author if you are interested in such data.

## Abbreviations

GI: Gastrointestinal
GERD: Gastroesophageal reflux disease
NR: Not reported
